# Illusory visual motion stimulus elicits postural sway in migraine patients

**DOI:** 10.3389/fpsyg.2015.00542

**Published:** 2015-04-28

**Authors:** Shu Imaizumi, Motoyasu Honma, Haruo Hibino, Shinichi Koyama

**Affiliations:** ^1^Graduate School of Engineering, Chiba UniversityChiba, Japan; ^2^Japan Society for the Promotion of ScienceTokyo, Japan; ^3^School of Medicine, Showa UniversityTokyo, Japan; ^4^School of Mechanical and Aerospace Engineering, Nanyang Technological University, SingaporeSingapore

**Keywords:** migraine, vision, optical illusion, postural control, visuo-vestibular interaction, multisensory integration

## Abstract

Although the perception of visual motion modulates postural control, it is unknown whether illusory visual motion elicits postural sway. The present study examined the effect of illusory motion on postural sway in patients with migraine, who tend to be sensitive to it. We measured postural sway for both migraine patients and controls while they viewed static visual stimuli with and without illusory motion. The participants’ postural sway was measured when they closed their eyes either immediately after (Experiment 1), or 30 s after (Experiment 2), viewing the stimuli. The patients swayed more than the controls when they closed their eyes immediately after viewing the illusory motion (Experiment 1), and they swayed less than the controls when they closed their eyes 30 s after viewing it (Experiment 2). These results suggest that static visual stimuli with illusory motion can induce postural sway that may last for at least 30 s in patients with migraine.

## Introduction

Postural control is modulated not only by vestibular functioning (Birren, 1945) but also by visual stimulation. For example, visual input simulating forward or backward self-motion, such as expanding, or contracting optic flow, elicits postural sway in observers (Lee and Lishman, 1975; van Asten et al., 1988). This visually induced postural modulation occurs even in infants (Lee and Aronson, 1974). These and other recent studies (Guerraz and Bronstein, 2008; Meyer et al., 2013) suggested that postural sway was induced by the visual stimulus with motion energy (i.e., a physically moving stimulus).

**FIGURE 1:**
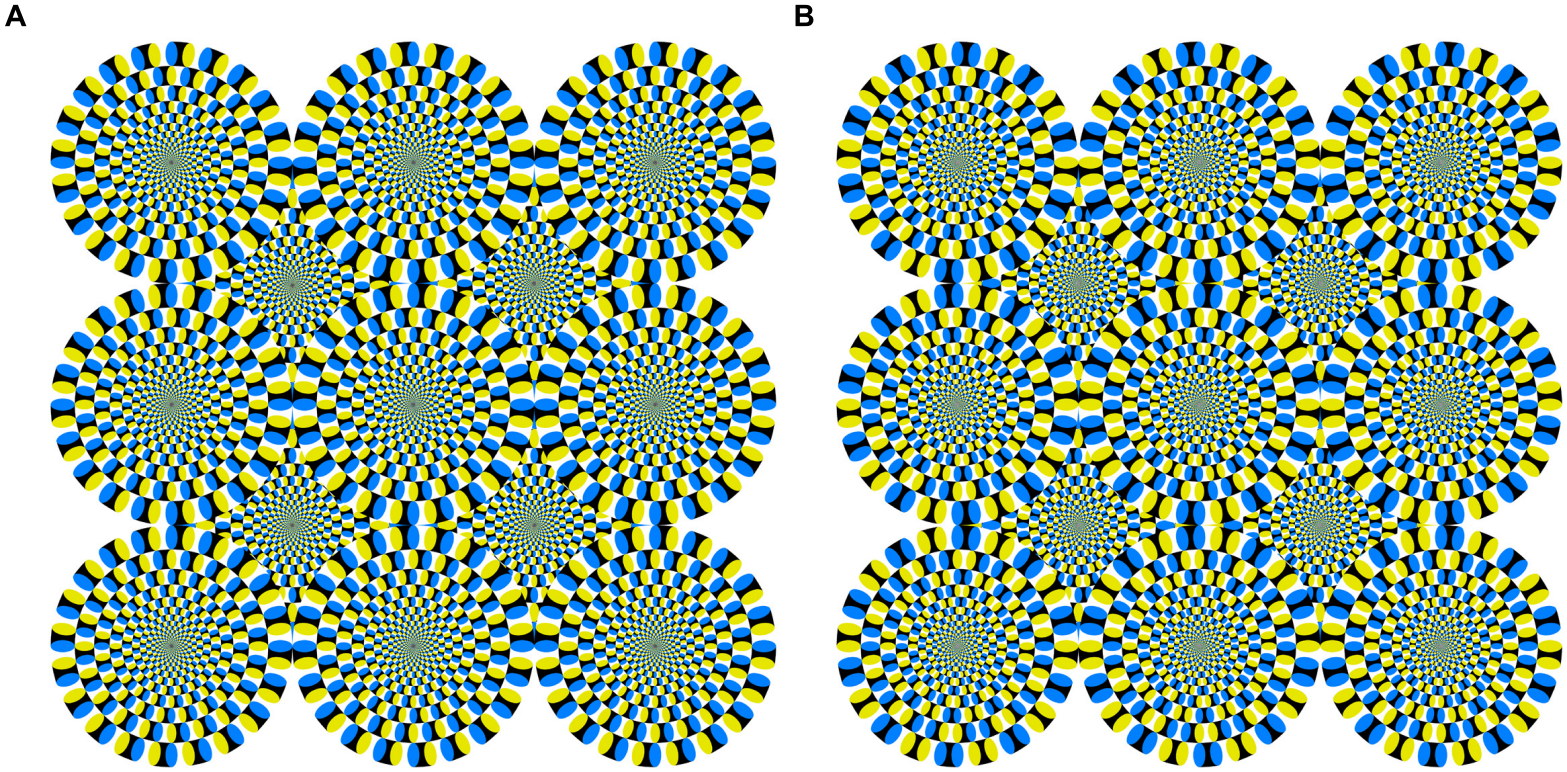
**(A)** Snake image with illusory motion used in Experiments 1 and 2, and **(B)** reversed image without illusory motion used in Experiment 2. Both were reproduced with permission from the author (Kitaoka, 2011).

However, human observers do not necessarily need motion energy to perceive motion in a visual stimulus. Illusory motion perception is one type of optical illusion in which observers perceive physically static images as moving. In the Fraser–Wilcox illusion (Fraser and Wilcox, 1979), a static figure consisting of repeating patterns with saw-tooth luminance profiles induces illusory motion. The Rotating Snakes (Kitaoka, 2003) is an optimized Fraser–Wilcox illusion that has patterns with stepwise luminance profiles, which induces stronger illusory motion (Kitaoka and Ashida, 2003; See **Figure 1A** for an example). One explanation for the Rotating Snakes is that each component of the stepwise luminance profiles in this figure elicits motion energy caused by differences in the latency of neural activity for each luminance component (Backus and Oruc, 2005; Conway et al., 2005). Recent studies have suggested that the neural basis for the illusory motion induced by Rotating Snakes is found in the human cortical pathway from primary visual cortex to the middle temporal area (Kuriki et al., 2008; Ashida et al., 2012).

The effects of illusory motion on human body movements are not well documented. One previous study reported that illusory expanding motion can induce the perception of body movement in physically stationary observers (i.e., forward vection; Seno et al., 2013). Another study showed that, following adaptation to a leftward or rightward 20-s random-pixel array motion, the aftereffect resulting from the static random pixels increased postural sway in the direction opposite to that of the adapted motion (Holten et al., 2014). The researchers argued that the neural motion signal itself influences postural control, even after moving stimulus observation. Indeed, physically moving visual stimulation has been demonstrated to activate the visual cortex’s middle temporal area (Zeki et al., 1991; Morrone et al., 2000), which can also be activated by illusory motion (Kuriki et al., 2008; Ashida et al., 2012) and motion aftereffect (He et al., 1998). On the other hand, an optical flow stimulus that is congruent with self-motion can activate not only the middle temporal area (Slobounov et al., 2006), but also the cingulate sulcus visual area, which receives vestibular inputs (Smith et al., 2012) and represents self-motion (Wall and Smith, 2008; Fischer et al., 2012). Since physical motion perception shares common neural bases with illusory motion and motion aftereffect and is represented in the self-motion sensitive cortex, illusory motion may influence postural control, as well as physical motion (e.g., Lee and Lishman, 1975) and motion aftereffect (Holten et al., 2014). However, it remains unclear whether postural sway increases *during* the illusory motion inducing static visual stimulus observation.

Illusory motion and/or visual distortion in static geometrical stimuli (e.g., striped patterns) are more likely to be perceived by individuals with chronic migraine headaches than non-chronic headache sufferers (Wilkins et al., 1984; Marcus and Soso, 1989; Huang et al., 2003; Imaizumi et al., 2011). This effect is perhaps caused by altered cortical processing in the primary visual cortex (Aurora et al., 1998; Huang et al., 2003) and middle temporal areas (Granziera et al., 2006). On the other hand, migraine patients are known to be susceptible to motion sickness (Cutrer and Baloh, 1992; Drummond, 2005; Marcus et al., 2005), which is caused by the conflict between visual and vestibular input (Reason and Brand, 1975; Yates et al., 1998). Especially in patients with migraine, motion sickness can be evoked solely by visual stimulation when it conflicts with vestibular signals. For instance, the stationary observation of horizontally moving vertical stripes can induce motion sickness more in patients than in normal controls (Drummond, 2002; Drummond and Granston, 2004). Thus, it can be assumed that patients with migraine, who are susceptible to visually induced motion sickness, might be more dependent on visual input when their posture is controlled. Although postural sway increases in both patients and normal controls when they close their eyes because of the lack of visual control (Travis, 1945; Edwards, 1946; Honma et al., 2012), a previous study demonstrated that postural sway increases by a greater amount in patients with migraine while they have their eyes closed (Ishizaki et al., 2002). Taken together, we hypothesize that the patients’ postural control should be more influenced by visual stimuli than that of normal individuals, especially when the stimuli are capable of inducing illusory motion.

The present study aimed to examine whether illusory motion can influence postural sway and whether there are any distinguishing characteristics in patients with migraine in terms of postural control. We attempted to measure the postural sway of both patients and normal controls during observations of static visual stimuli with and without illusory motion with a stabilometer to track the displacement of centers of gravity.

## Experiment 1

We measured postural sway during migraine patients’ and normal controls’ viewing of static stimulus with and without illusory motion (Rotating Snakes and a gray plane, respectively).

### Materials and Methods

#### Participants

This experiment included 11 patients with migraine (six female; mean age 22.18 ± 0.30 years) and nine controls without chronic headaches (two female; mean age 22.22 ± 0.40 years). One of the patients had visual aura symptoms. We separated the patients from the controls and determined the presence of visual aura using a questionnaire based on the second edition of the International Classification of Headache Disorders (Headache Classification Subcommittee of the International Headache Society, 2004), which includes 18 questions about chronic headache occurrence, as well as their characteristics, duration, frequency, and accompanying symptoms. All participants had normal or corrected-to-normal visual acuity with no visual deficits, such as color blindness. The experiment was conducted during headache-free periods. Written informed consent was obtained from each participant. This study was approved by the ethical committee of the Graduate School of Engineering, Chiba University, and was conducted in accordance with the principles of the Declaration of Helsinki.

#### Apparatus

**FIGURE 2:**
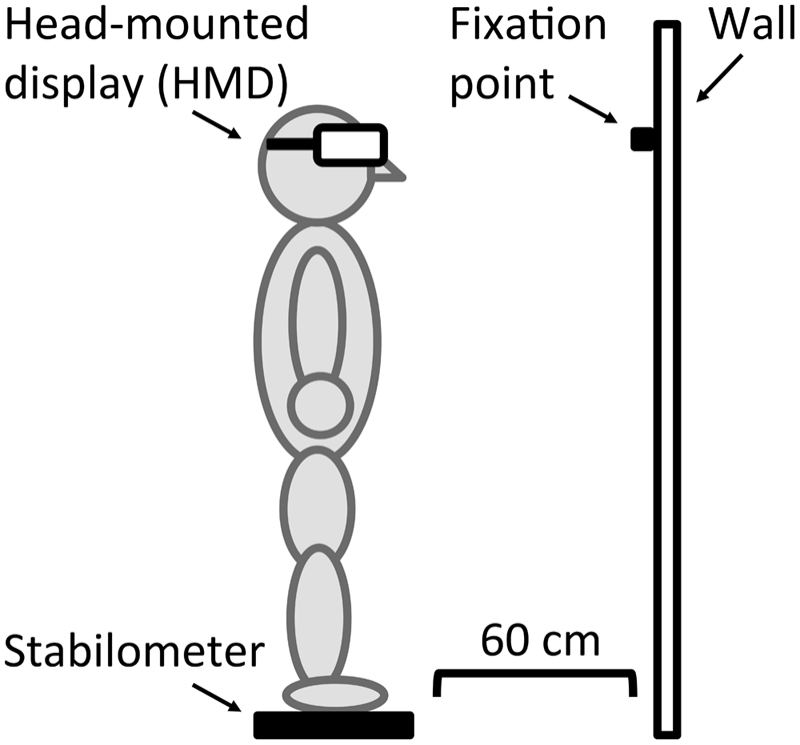
Apparatus used in Experiments 1 and 2.

**Figure 2** shows an example of the apparatus. Stimuli were presented on a HMD, (HMZ-T1, Sony Corporation). The luminance output from the HMD ranged from 0.40 to 28.36 cd/m^2^. A stabilometer (UM-BAR2, Unimec Corporation), which was placed on the floor 60 cm away from the wall, tracked participants’ centers of gravity displacements and sampled their fluctuations at 60 Hz.

#### Stimuli

We used two static visual stimuli: a homogeneous gray plane and an illusory motion image (Rotating Snakes Kitaoka, 2003, 2011). The illusory motion image (the “snake image”), as shown in **Figure 1A**, has been used in many motion perception studies (Conway et al., 2005; Kuriki et al., 2008; Ashida et al., 2012). The smallest unit of the snake image composition was an arrangement of “black–blue–white–yellow” patches. This color patch order was arranged in the same direction throughout, thus inducing illusory rotational motion. Each stimulus included a fixation cross at its center. All had the same mean luminance of 13.56 cd/m^2^ and were subtended at approximately 29 by 29° on the HMD’s black background.

#### Procedure

Our procedure followed a standard stabilometric protocol based on Kapteyn et al. (1983) and Ishizaki et al. (2002), who investigated postural control in patients with migraine. The participants removed their shoes and stood erect, with their knees straight and hands down at their sides, on the stabilometer. First, they stood on the stabilometer without HMD and viewed an eye-level fixation point on the wall for 30 s (eyes open condition). Immediately afterward, they closed their eyes and kept standing for 30 s (eyes closed condition). Next, they stood on the stabilometer with the HMD on their heads and fixated on the center cross on one of the two stimuli for 30 s. Then, they closed their eyes and kept standing on the stabilometer for 30 more seconds. The stimuli were presented in a random order. These procedures were the same across three trials (one per condition). The number of trials was limited in order to prevent excessive visual stress (Wilkins, 1995), such as eye strain and visual discomfort, and to reduce the risk of migraine attacks (Harle et al., 2006).

We recorded the stabilometric parameters of postural sway, total path length (total length of center-of-gravity displacement), rectangular area (area of the maximum amplitude of center-of-gravity displacement), and Romberg ratio (postural sway parameter ratio of measurement under the eyes closed condition to that of the eyes open condition). The Romberg ratio assesses the stabilizing effect of vision in postural control (Diener et al., 1984) and typically measures more than 1 because one’s postural sway tends to increase when one’s eyes are closed (Travis, 1945; Edwards, 1946; Honma et al., 2012).

#### Data Analysis

Total path length (eyes open and closed condition, and its Romberg ratio) and rectangular area (eyes open and closed condition, and its Romberg ratio) were independently analyzed using RMANOVA with a between-participants factor (migraine: patients, controls) and a within-participants factor (stimulus type: without HMD, gray plane, snake image). Because of our relatively small sample size, we did not analyze the effect of the presence of visual aura, although migraine with aura has been suggested to be associated with strong perceptual disturbances (Chronicle and Mulleners, 1994; Shepherd, 2000; Cucchiara et al., 2014). When the sphericity assumption of the RMANOVA was violated, Greenhouse–Geisser correction was applied to the degrees of freedom. Bonferroni correction was used for multiple comparisons. The significance level was set at *p* < 0.05. The effect size was reported as eta squared (η^2^).

### Results

**FIGURE 3:**
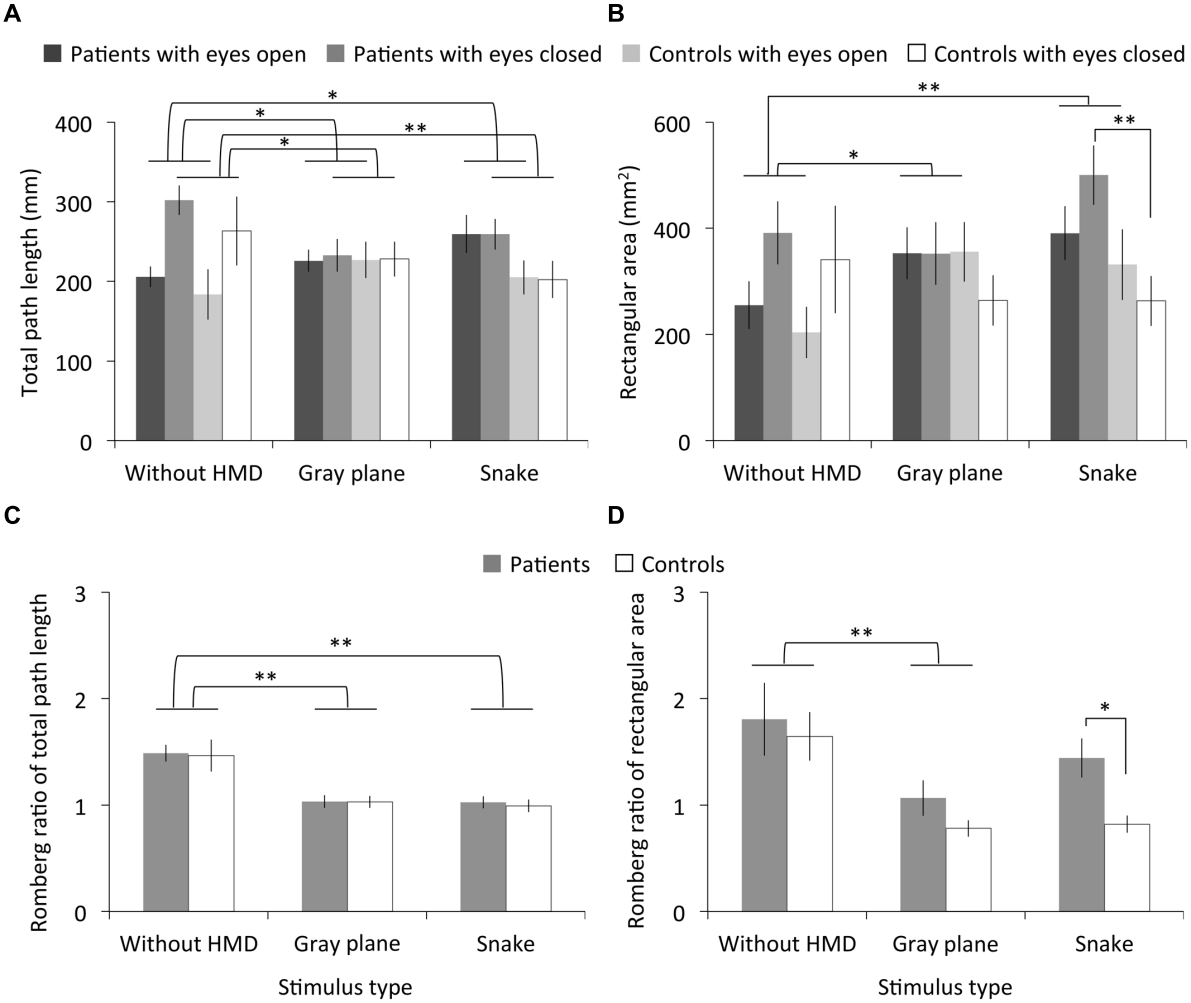
Results of Experiment 1. **(A)** Total path length, **(B)** rectangular area, and **(C,D)** their Romberg ratios for patients with migraine and normal controls as a function of stimulus type. Error bars denote ± 1 SEM. Asterisks indicate significant differences (^∗^*p* < 0.05, ^∗∗^*p* < 0.01).

**Figure 3** shows the measured total path length, rectangular area, and their Romberg ratio of both the migraine patients and controls. The RMANOVA revealed significant main effects of stimulus type on total path length under the eyes open and closed conditions and on the Romberg ratio of the total path length [eyes open: *F*_(2,36)_ = 4.48, *p* < 0.05, η^2^ = 0.20; eyes closed: *F*_(2,36)_ = 7.16, *p* < 0.01, η^2^ = 0.29; Romberg ratio: *F*_(1.50,27.08)_ = 19.69, *p* < 0.01, η^2^ = 0.52]. Multiple comparisons revealed significantly larger total path length in the eyes open condition with the gray plane and snake image than in the without HMD condition (*p*s < 0.05) and smaller total path length in the eyes closed condition and its Romberg ratio with the gray plane and snake image (*p*s < 0.01; except for the eyes closed with gray plane condition: *p* < 0.05). We found no significant main effects of migraine or interaction between migraine and stimulus type on total path length and the Romberg ratio of total path length [migraine on eyes open condition: *F*_(1,18)_ = 0.95, *p* = 0.34, η^2^ = 0.05; eyes closed: *F*_(1,18)_ = 1.23, *p* = 0.28, η^2^ = 0.06; Romberg ratio: *F*_(1,18)_ = 0.10, *p* = 0.76, η^2^ = 0.01; interaction on eyes open condition: *F*_(2,36)_ = 2.14, *p* = 0.13, η^2^ = 0.11; eyes closed: *F*_(2,36)_ = 1.39, *p* = 0.26, η^2^ = 0.07; Romberg ratio: *F*_(1.50,27.08)_ = 0.01, *p* = 0.97, η^2^ = 0.00].

On the other hand, we found significant main effects of stimulus type on rectangular area under the eyes open condition and for the Romberg ratio of the rectangular area [eyes open: *F*_(2,36)_ = 8.52, *p* < 0.01, η^2^ = 0.32; Romberg ratio: *F*_(2,36)_ = 7.65, *p* < 0.01, η^2^ = 0.30] but not on rectangular area under the eyes closed condition [*F*_(2,36)_ = 1.26, *p* = 0.30, η^2^ = 0.07]. Multiple comparisons revealed significantly larger rectangular area in the eyes open condition with the gray plane (*p* < 0.05) and snake image (*p* < 0.01) than in the without HMD condition and a smaller Romberg ratio of total path length with the gray plane, although there was no main effect of stimulus type on Romberg ratio (*p* < 0.01). We found no significant main effects of migraine or interaction between migraine and stimulus type on the rectangular area and the Romberg ratio of the rectangular area [migraine on eyes open condition: *F*_(1,18)_ = 0.34, *p* = 0.57, η^2^ = 0.02; eyes closed: *F*_(1,18)_ = 3.15, *p* = 0.09, η^2^ = 0.15; Romberg ratio: *F*_(1,18)_ = 4.17, *p* = 0.06, η^2^ = 0.19; interaction on eyes open condition: *F*_(2,36)_ = 0.44, *p* = 0.65, η^2^ = 0.02; eyes closed: *F*_(2,36)_ = 2.05, *p* = 0.14, η^2^ = 0.10; Romberg ratio: *F*_(2,36)_ = 0.63, *p* = 0.54, η^2^ = 0.03]. However, multiple comparisons revealed a significantly larger rectangular area in patients compared to controls in the eyes closed condition after the observation of the snake image (*p* < 0.01). Consequently, the patients’ Romberg ratio of the rectangular area was significantly higher (*p* < 0.05).

### Discussion

No differences in total path length were found between the gray plane and the snake image observations, while the total path length under the without HMD condition increased more than that under the gray plane and snake image eyes open conditions and their Romberg ratios. This is the case concerning the rectangular area, except for the Romberg ratio in the without HMD and snake image conditions. Participants likely increased their postural sway during the observation of both the gray plane and the snake image. Postural sway may be elicited by visual stimulation with HMD, regardless of illusory motion (Hakkinen et al., 2002).

Concerning the differences between participants, there were no total path length differences between the migraine patients and controls. However, the patients showed larger rectangular area while closing their eyes after viewing the illusory rotating snake image, whereas such differences were not found during the actual observation. There are three possible explanations for these results. First, since migraine patients perceive stronger motion aftereffects than controls (Shepherd, 2006), the illusory motion aftereffect may have increased the patients’ postural sway. Indeed, postural sway can be elicited by the motion aftereffect following continuous observations of a horizontally moving visual stimulus (Holten et al., 2014). An alternative hypothesis is that visual stress *per se* induced postural sway. Migraine patients are known to be particularly susceptible to striped patterns with unnatural characteristics (Fernandez and Wilkins, 2008; Juricevic et al., 2010; Penacchio and Wilkins, 2015), and such visual patterns are likely to evoke excess visual cortex excitation (Huang et al., 2003, 2011). Because our snake image contained visual patterns similar to high-contrast stripes, they might have induced the non-specific visual disturbance and the visual pattern cortical response, which would induce postural sway even after the eyes were closed. Finally, migraine patients may simply be more susceptible to sway with closed eyes (Ishizaki et al., 2002). To test these hypotheses, we carried out another experiment including a 30-s interval between the eyes open and closed conditions. If the patients’ sway during the eyes closed condition is induced by motion aftereffect or visual stress, the effect will be reduced after the 30-s interval.

## Experiment 2

To examine whether the illusory motion-generated aftereffect can increase postural sway, we inserted an interval between the eyes open and closed conditions to decay the aftereffect. The aftereffect decay should decrease postural sway. Furthermore, we used a snake image without illusory motion as a control stimulus (i.e., one that looked like the Rotating Snakes without the rotating effect; **Figure 1B**). If illusory motion is enough to modulate postural sway, then the control stimulus should not have the same effect.

### Materials and Methods

The material and methods were identical to those used in Experiment 1, except as noted below.

#### Participants

This experiment included eight patients with migraine (four female; mean age 21.29 ± 3.09 years) and 14 controls without chronic headaches (seven female; age 22.36 ± 2.24 years) who did not participate in Experiment 1. Two of the patients had visual aura symptoms.

In this experiment, we attempted to investigate migraine patients’ motion sickness susceptibility, since this is a common complaint among this population (e.g., Cutrer and Baloh, 1992) and is associated with visually induced postural instability in individuals highly susceptible to motion sickness (Smart et al., 2002; Yokota et al., 2005). According to a standardized questionnaire (Golding, 1998), patients and controls had compatible motion sickness susceptibility (patients: mean = 54.88, SD = 38.15; controls: mean = 53.06, SD = 30.20; *t*_(20)_ = 0.12, *p* = 0.90, Cohen’s *d* = 0.05). The patients showed slightly low, and controls showed high, scores in comparison with Jeong et al. (2010), who investigated migraine patients’ abnormal vestibular functions of migraine patients (patients: approximately 59; controls: approximately 38. Note they reported only graphs without detailed values).

#### Stimuli

We used three stimuli: the gray plane and snake image used in Experiment 1 and a reversed image without illusory motion (Kitaoka, 2011) as a control stimulus (**Figure 1B**). The color patch order in the reversed image was reversed between adjacent units to nullify the illusory motion signal. Each stimulus included a fixation cross at its center. All had the same mean luminance of 13.56 cd/m^2^ and were subtended at approximately 29 by 29° on the HMD’s black background.

#### Procedure

To prevent the illusory motion-generated aftereffect from modulating postural sway in the eyes closed condition, we added intervals of 30 s between the eyes open and closed conditions for each measurement. During this interval, the participants who had their eyes open kept standing on the stabilometer while being exposed to a blank display for 30 s. They then closed their eyes, and their postural-sway indices were measured under the eyes closed condition. Directly after the stabilometric measurements, the participants orally rated the magnitude of illusory motion for each stimulus using an 11-point Likert scale, where 0 meant “the image did not appear to move at all,” and 10 meant “the image appeared to move most strongly.” These procedures were the same across four trials (one per condition).

As in Experiment 1, we conducted only a few trials in order to prevent excessive visual stress and reduce migraine attack risk. For the same reason, we decided not to conduct another trial for measuring the magnitude of illusory motion. Instead, we asked participants to report the perceived illusory motion retrospectively.

#### Data Analysis

Along with total path length and rectangular area, the illusory motion ratings were analyzed using RMANOVA with a between-participants factor (migraine) and a within-participants factor (stimulus type: without HMD, gray plane, snake image, reversed image).

### Results

**FIGURE 4:**
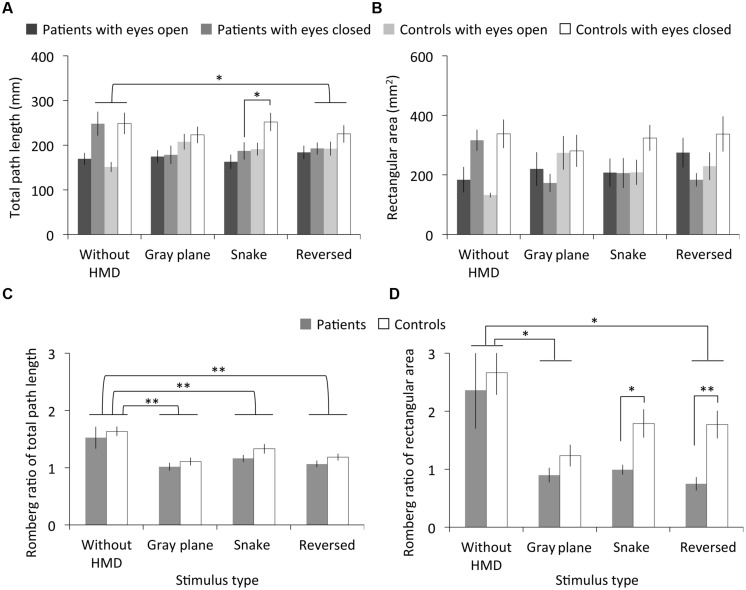
Results of Experiment 2. **(A)** Total path length, **(B)** rectangular area, and **(C,D)** their Romberg ratios for patients with migraine and normal controls as a function of stimulus type. Error bars denote ±1 SEM. Asterisks indicate significant differences (^∗^*p* < 0.05, ^∗∗^*p* < 0.01).

**Figure 4** shows the measured total path length, rectangular area, and Romberg ratio of both the patients and controls. The RMANOVA revealed significant main effects of stimulus type on total path length under the eyes open and closed conditions and on the Romberg ratio of total path length [eyes open: *F*_(2.24,44.70)_ = 4.16, *p* < 0.05, η^2^ = 0.17; eyes closed: *F*_(1.99,39.87)_ = 4.68, *p* < 0.05, η^2^ = 0.19; Romberg ratio: *F*_(3,60)_ = 15.43, *p* < 0.01, η^2^ = 0.44]. Multiple comparisons revealed significantly smaller total path length in the eyes closed condition after the observation of the reversed image than in the without HMD condition (*p* < 0.05), and a smaller Romberg ratio of total path length with the gray plane, snake, and reversed images than was observed in the without HMD conditions (*p*s < 0.01). We found no significant main effects of migraine or interaction between migraine and stimulus type on total path length and the Romberg ratio of total path length [migraine on eyes open condition: *F*_(1,20)_ = 0.49, *p* = 0.49, η^2^ = 0.02; eyes closed: *F*_(1,20)_ = 1.78, *p* = 0.20, η^2^ = 0.08; Romberg ratio: *F*_(1,20)_ = 2.33, *p* = 0.14, η^2^ = 0.10; interaction on eyes open condition: *F*_(2.24,44.70)_ = 2.61, *p* = 0.08, η^2^ = 0.12; eyes closed: *F*_(1.99,39.87)_ = 1.96, *p* = 0.15, η^2^ = 0.09; Romberg ratio: *F*_(3,60)_ = 0.08, *p* = 0.97, η^2^ = 0.00]. However, multiple comparisons revealed a significantly smaller total path length in patients compared to controls in the eyes closed condition after the snake image observation (*p* < 0.05).

There were no significant main effects of stimulus type and migraine or interaction between migraine and stimulus type on rectangular area under the eyes open and closed conditions [stimulus type on eyes open condition: *F*_(2.27,45.43)_ = 2.54, *p* = 0.08, η^2^ = 0.11; eyes closed: *F*_(2.34,46.74)_ = 2.35, *p* = 0.10, η^2^ = 0.11; migraine on eyes open condition: *F*_(1,20)_ = 0.05, *p* = 0.83, η^2^ = 0.00; eyes closed: *F*_(1,20)_ = 3.10, *p* = 0.09, η^2^ = 0.13; interaction on eyes open condition: *F*_(2.27,45.43)_ = 0.81, *p* = 0.49, η^2^ = 0.04; eyes closed: *F*_(2.34,46.74)_ = 1.07, *p* = 0.37, η^2^ = 0.05]. However, although no main effect of stimulus type was found, multiple comparisons revealed a significantly smaller Romberg ratio for the rectangular area with the gray plane and reversed image than was observed in the without HMD condition (*p*s < 0.05). On the other hand, we found significant main effects of stimulus type and migraine on the Romberg ratio of rectangular area [stimulus type: *F*_(1.48,29.57)_ = 8.57, *p* < 0.01, η^2^ = 0.30; migraine: *F*_(1,20)_ = 7.56, *p* < 0.05, η^2^ = 0.27], but no significant interactions between these factors [*F*_(1.48,29.57)_ = 0.63, *p* = 0.60, η^2^ = 0.03]. Contrary to Experiment 1’s results, multiple comparisons revealed that the Romberg ratio of the rectangular area significantly decreased in patients relative to controls following both the snake (*p* < 0.05) and reversed image observations (*p* < 0.01).

**FIGURE 5:**
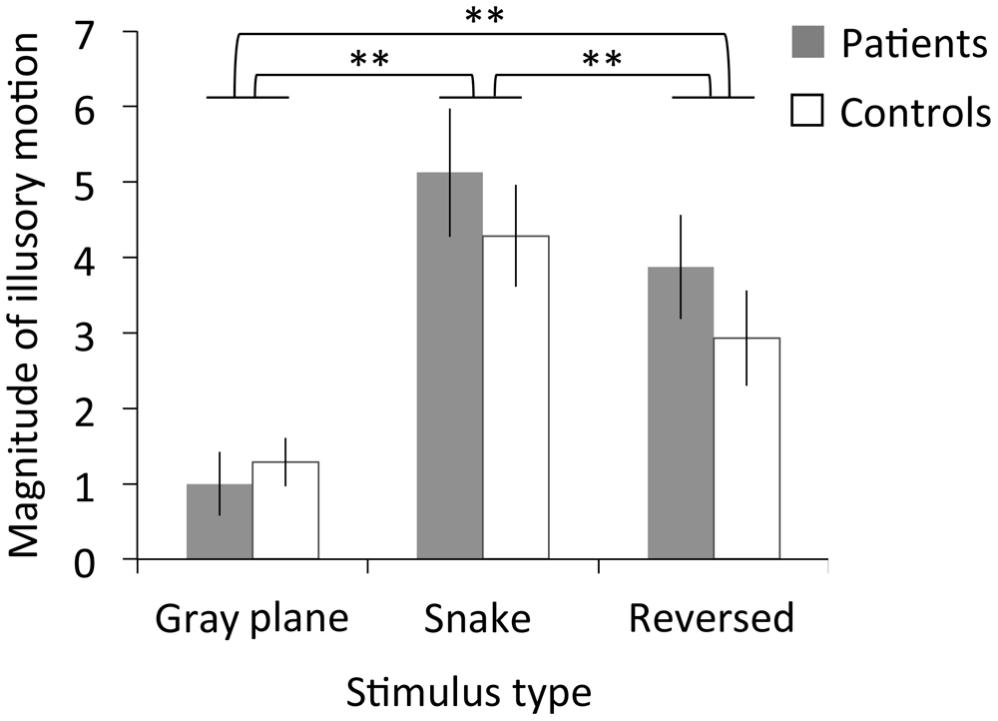
Results of Experiment 2. Magnitude ratings for illusory motion in patients with migraine and normal controls as a function of stimulus type. Error bars denote±1 SEM. Asterisks indicate significant differences (^∗∗^*p* < 0.01).

**Figure 5** depicts the subjective magnitude of illusory motion for both the patients and controls. The RMANOVA revealed significant main effects of stimulus type on magnitude [*F*_(2,40)_ = 24.53, *p* < 0.01, η^2^ = 0.55]. No significant main effects of migraine or interaction between migraine and stimulus type were found [migraine: *F*_(1,20)_ = 0.53, *p* = 0.48, η^2^ = 0.03; interaction: *F*_(2,40)_ = 0.88, *p* = 0.42, η^2^ = 0.04]. Multiple comparisons revealed that illusory motion significantly increased for the snake image relative to both the gray plane and the reversed image, and for the reversed image relative to the gray plane (*p*s < 0.01).

### Discussion

The results showed differences in total path length and rectangular area Romberg ratios between the without HMD condition and each of the three visual stimuli conditions, except for the Romberg ratio of rectangular area with the snake image, while no differences were found among the stimuli. Similar to Experiment 1’s findings, postural sway in both patients and controls was apparently elicited by visual stimulation with HMD, regardless of illusory motion (Hakkinen et al., 2002).

There were no total path length differences between migraine patients and controls, except for longer total path length among the controls under the eyes closed condition after snake image observation. However, contrary to Experiment 1’s results, a smaller Romberg ratio for the migraine patients suggested they showed decreased postural sway in the eyes closed condition after observing both the snake and reversed images following a 30-s interval. Therefore, an illusory motion-generated aftereffect can increase postural sway in migraine patients.

Visual stress and discomfort due to stimulus spatial properties (e.g., Fernandez and Wilkins, 2008) can also explain the increased postural sway following observation. Although the snake image created stronger illusory motion than did the reversed image for both patients and controls, there were no significant differences between the patients’ postural sway for either image. In addition, the reversed image also induced more illusory motion than did the gray plane, suggesting that the geometric repetitive patterns of the reversed image may have induced perceptual distortions in migraine patients and controls as a consequence of neural overload (Wilkins, 1995; Imaizumi et al., 2011).

## General Discussion

The present study investigated how migraine patients’ postural sway can be modulated by static visual stimuli, especially stimuli with illusory motion perception. In Experiment 1, patients showed larger sway while closing their eyes after viewing the illusory motion. In Experiment 2, they showed decreased sway while closing their eyes after a 30-s interval following their viewing of the illusory motion. Thus, static visual stimuli can induce illusory motion and postural sway, and this effect may last for at least 30 s among the patients.

We hypothesized two mechanisms underlying the increased sway in patients with migraine who closed their eyes after viewing the illusory motion. First, due to their sensitivity to the illusory motion (Huang et al., 2003; Imaizumi et al., 2011) and/or motion aftereffect (Shepherd, 2006), the motion aftereffect continued even after the patients closed their eyes, and this induced postural sway. Although this finding is speculative due to a lack of evidence for the occurrence of aftereffects in the patients, recent findings suggesting that the motion aftereffect itself can induce postural sway (Holten et al., 2014) may support this hypothesis. Second, visual stress in the patients with migraine, which was caused by the stimuli (Wilkins, 1995; Huang et al., 2003, 2011), resulted in the propagation of the visual activities to the more anterior motion- and vestibular-related areas. Consequently, these abnormal neural responses may have induced postural sway due to perceptual disturbances that last for 30 s after the stimulus observation. Given that high-contrast stripes with unnatural spatial characteristics, in terms of the Fourier amplitude spectrum of images (Fernandez and Wilkins, 2008; Juricevic et al., 2010; O’Hare and Hibbard, 2011; Penacchio and Wilkins, 2015), can evoke visual stress (Huang et al., 2003, 2011), our snake and reversed images with patterns similar to high-contrast stripes might have induced the visual stress-induced sway. Such postural sway could be found in both patients and controls, because visual stress is not limited to migraine patients. Normal individuals also find some images uncomfortable to view (Conlon et al., 1999; Fernandez and Wilkins, 2008). However, no studies have reported how long, and to what extent, visual stress can influence postural control when one’s eyes are closed. Future studies testing these hypotheses should be beneficial in understanding vision, postural control, and their interactions, especially in migraine patients.

Moreover, we found differences between migraine patients and controls, mostly in the rectangular area. Generally, rectangular area reflects how widely, whereas total path length reflects how frequently the centers of pressure fluctuate. Therefore, patients’ greater postural sway as induced by the visual stimuli with illusory motion can appear widely and slowly after their eyes closed. This characteristic of sway is consistent with Ishizaki et al. (2002), who reported that patients with eyes closed showed larger rectangular area than normal controls but no total path length differences between them, although they did not examine the effect of visual stimulation.

However, it is unclear why our participants did not show more postural sway during their illusory motion observations. There are three possible explanations. First, although visual stimuli with illusory motion may elicit perceptions of body movement (Seno et al., 2013), such stimuli may not lead to actual body movement (i.e., postural sway), which suggests postural sway can be modulated only by direct visual-motion stimulation. Second, HMD weight (∼420 g) itself may have caused posture-controlling difficulties, thus attenuating the conditions’ effects on postural sway. Indeed, postural instability during an observation with HMD may occur more strongly than that occurring during television viewing (Hakkinen et al., 2002). Finally, negative emotional processes may have influenced postural control. Postural sway can be decreased by visually evoked negative emotions such as disgust (Azevedo et al., 2005; Stins and Beek, 2007), and by imagined painful situations (Lelard et al., 2013), suggesting the activation of a defensive “freezing” posture. Our results showing no increased postural sway during the snake image observation may indicate that visual discomfort cancels out postural sway during observation of the illusory motion stimuli, even though we did not measure perceived visual discomfort. Further investigations should overcome the abovementioned methodological issues by manipulating emotional components in illusory motion stimuli to clarify the effects of illusory motion and visual discomfort on postural sway in light of migraine patients’ perceptual characteristics.

Although the two experimental procedures were identical except for the trial number and the 30-s interval between the eyes open and eyes closed measurements, the results obtained from the two experiments seem to differ in several ways besides the illusory motion aftereffect, as noted above. Decreased sway *during* the stimulus observation was found in Experiment 2, although the presence of the 30-s intervals should affect postural sway *after* the observation. We speculate that inter-individual variability in visually induced postural sway (Akiduki et al., 2003), in addition to the migraine effect, may have led to such inter-experiment differences, given that all participated in either Experiment 1 or 2. Besides, motion sickness susceptibility might be the potential factor in increasing postural sway, since visually induced postural instability can be found in highly susceptible individuals (Smart et al., 2002; Yokota et al., 2005); however, there is lack of susceptibility evidence from Experiment 1’s participants.

The present study has several limitations. First, the illusory rotating motion parallel to the coronal plane induced by the snake image did not allow us to examine how illusory motion direction and magnitude were associated with those of postural sway, although the perceived motion direction will be consistent with the direction of increased sway (Lee and Lishman, 1975; Bronstein, 1986). Furthermore, the illusory rotation of one part of the snake image might be counterbalanced by the opposite rotation of another part. If this is the case, we can speculate that overall rotation decreased and, consequently, did not elicit postural sway in the specific direction. Indeed, a follow-up analysis revealed that the ratio of medio-lateral to antero–posterior path length did not differ among stimuli for patients and controls in either Experiment (no main effects of stimulus type: *F*s < 3.91, *p*s > 0.06, η^2^s < 0.17; no main effects of migraine: *F*s < 0.11, *p*s > 0.74 η^2^s < 0.01). This suggests that our stimuli that included the illusory motion stimulus influenced the amount of postural sway but did not bias the direction of the sway. As Holten et al. (2014) used the horizontally moving stimuli in the coronal plane, further investigation is needed to clarify the direction and magnitude of sway induced by illusory motion in the antero–posterior and medio-lateral dimensions. Second, we measured only one trial for each experimental condition in order to avoid excessive visual stress and the risk of migraine attacks being triggered by visual stimuli (Wilkins, 1995; Harle et al., 2006), resulting from long-term exposure to the illusory motion stimuli, in particular. Finally, we did not measure the perceived illusory motion *during* the stimulus presentation. Instead, we measured this *after* the presentation and limited the number or trials for the abovementioned ethical reason. However, given that there is large inter-individual variability in postural sway (Akiduki et al., 2003) and probable inaccuracy of retrospective perceptual judgment, future studies with larger sample sizes and adequate inter-trial intervals will allow for the repeated measurement of postural sway and separate sessions with which to measure illusory motion more accurately.

In conclusion, the present study examined how illusory motion influenced postural sway in migraine patients and normal controls. We proposed the possibility that illusory motion and visual stress may induce postural sway in migraine patients after illusory motion stimulus observation, although we could not dissociate their effects. Future studies are required to confirm this possibility, considering the multiple factors associated with vision and postural control in migraine patients, such as motion sickness susceptibility and visual discomfort.

## Conflict of Interest Statement

The authors declare that the research was conducted in the absence of any commercial or financial relationships that could be construed as a potential conflict of interest.

## ABBREVIATIONS

HMD: head-mounted display
RMANOVA: repeated measures analysis of variance.
